# THE IMPACT OF MORTALITY SALIENCE ON COLLEGE STUDENTS’ INTENT TO HELP OLDER ADULTS

**DOI:** 10.1093/geroni/igz038.311

**Published:** 2019-11-08

**Authors:** Erika A Fenstermacher, Jessica Birg, Vincent Barbieri, Nathaniel Herr

**Affiliations:** American University, Washington, District of Columbia, United States

## Abstract

Terror Management Theory (TMT) states that the awareness of one’s own death causes humans to experience intense anxiety, which must be continuously managed. Much of the research on TMT has focused on negative outcomes, rather than prosocial behavior, begging the question: “Can priming individuals with the thought of their own death trigger them to behave in ways that benefit others?”. Jonas et al. (2002), found that when mortality salience was primed prosocial behavior increased. In line with TMT, they hypothesized that people may behave in a more prosocial manner as it fits in with their personal values. The present study recruited 108 students who were randomly assigned to a mortality salience (MS) or control condition. Participants also completed baseline self-reports, which included measures of ageism, social desirability, personality, and empathy. After the study seemed to end, participants were given a disguised measure of helping behavior, which they believed to be an interest survey for a student volunteer group. Preliminary analyses indicate that those in the MS condition were more willing to be contacted to volunteer with kids than being contacted to volunteer with older adults. We also found that those in the MS condition were more likely to be contacted to volunteer with kids than those in the control condition. Our findings are consistent with previous work showing that individuals favor their ingroup when primed with their death. This reflects the importance of focused efforts on encouraging young people to identify with older adults and on promoting prosocial behavior.

